# Detection and relevance of epigenetic markers on ctDNA: recent advances and future outlook

**DOI:** 10.1002/1878-0261.12978

**Published:** 2021-05-14

**Authors:** Evi Lianidou

**Affiliations:** ^1^ Analysis of Circulating Tumor Cells Laboratory of Analytical Chemistry Department of Chemistry University of Athens Greece

**Keywords:** cell‐free DNA, circulating tumor cells, circulating tumor DNA, DNA methylation, methylation‐specific PCR, minimal residual disease

## Abstract

Liquid biopsy, a minimally invasive approach, is a highly powerful clinical tool for the real‐time follow‐up of cancer and overcomes many limitations of tissue biopsies. Epigenetic alterations have a high potential to provide a valuable source of innovative biomarkers for cancer, owing to their stability, frequency, and noninvasive accessibility in bodily fluids. Numerous DNA methylation markers are now tested in circulating tumor DNA (ctDNA) as potential biomarkers, in various types of cancer. DNA methylation in combination with liquid biopsy is very powerful in identifying circulating epigenetic biomarkers of clinical importance. Blood‐based epigenetic biomarkers have a high potential for early detection of cancer since DNA methylation in plasma can be detected early during cancer pathogenesis. In this review, we summarize the latest findings on DNA methylation markers in ctDNA for early detection, prognosis, minimal residual disease, risk of relapse, treatment selection, and resistance, for breast, prostate, lung, and colorectal cancer.

## Graphical abstract

Blood‐based epigenetic biomarkers present in circulating tumor DNA (ctDNA) have a high potential for early detection of cancer, since DNA methylation in plasma can be detected early during cancer pathogenesis. In this review, we summarize the latest findings on DNA methylation markers in ctDNA for early detection, prognosis, minimal residual disease, risk of relapse, treatment selection, and resistance in patients with breast, prostate, lung, and colorectal cancer.

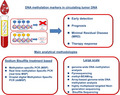

**Abbreviations**
AA: abiraterone acetate
BPH: benign prostatic hyperplasia
cfDNA: cell‐free DNA
CRC: colorectal cancer
CTCs: circulating tumor cells
ctDNA: circulating tumor DNA
ddMSP: droplet digital methylation‐specific PCR
ddPCR: droplet digital PCR
EMA: European Medicines Agency
FDA: Food and Drug Administration
mCRPC: metastatic castration‐resistant prostate cancer
MRD: minimal residual disease
MS‐ddPCR: methylation‐specific droplet digital PCR
MSP: methylation‐specific PCR
NSCLC: non‐small‐cell lung cancer
OS: overall survival
qMSP: quantitative methylation‐specific PCR
SB: sodium bisulfite
TCGA: Cancer Genome Atlas

## 1 Introduction

Liquid biopsy is a highly powerful clinical tool for the real‐time follow‐up of cancer patients that overcomes many limitations of tissue biopsies [1, 2]. Liquid biopsy approaches include the enumeration and molecular characterization of circulating tumor cells (CTCs), and the analysis of circulating tumor DNA (ctDNA), circulating miRNAs, and tumor‐derived extracellular vesicles that are shed from primary tumors and metastatic sites into peripheral blood. The major advantage of liquid biopsy analysis is that it is minimally invasive and can provide real‐time information on tumor characteristics in regular time intervals. ctDNA can be detected in the biological fluids of cancer patients as a small fraction of cell‐free DNA (cfDNA) that is usually detected at very low concentrations. ctDNA analysis is mainly focused on the detection of cancer‐specific mutations that are highly important for therapeutic treatment of and disease monitoring in cancer patients [3, 4, 5]. ctDNA analysis was recently shown to be highly promising for early cancer detection: CancerSEEK is a newly developed blood test that can detect eight common cancer types through the quantitation of circulating protein levels and mutations in cfDNA [6]. It is, however, important to point out that, although ctDNA analysis in most cases is targeted as it requires prior knowledge of the mutations to be analyzed, ctDNA in plasma is not only a subfraction of cfDNA but also a mixture of fragmented alleles derived from different cancer deposits.

Epigenetic alterations have a high potential to provide a valuable source of innovative biomarkers for cancer, since they are stable, highly frequent for specific genes, and can also be detected in biological fluids in a minimally invasive way. A variety of studies on DNA methylation markers focus on the evaluation of their clinical significance as novel epigenetic biomarkers [7]. Among epigenetic alterations, DNA methylation is very important in cancer since it affects gene expression in a similar way to how mutations affect gene functions [8]. The fact that DNA methylation is playing a crucial role in all types of cancer makes it ideal as a source of a variety of tumor biomarkers [9, 10].

Analysis of DNA methylation markers in primary tumors has already shown impressive results. The Cancer Genome Atlas (TCGA; https://cancergenome.nih.gov) is an important source of information for DNA methylation biomarkers in many types of cancer. In a recent study, homogenous groups of secondary breast cancers to matched cohorts of primary breast cancers that were included in the TCGA were compared in order to identify specific gene expression and DNA methylation signatures [11]. Significant differences were identified in gene expression and DNA methylation signatures in invasive ductal carcinomas and in invasive lobular carcinomas; these differences were found to be important for tumor growth and proliferation.

The characterization of molecular alterations specific for different disease subtypes is highly important [11]. A recent study focused on an integrative molecular analysis in all available TCGA tumor specimens in 33 different types of cancer; this study has shown that samples were clustered according to histology, type of tissue, and anatomic origin [12]. This study has clearly shown that clustering based on DNA methylation data was highly influenced by the cell type, emphasizing the important role of the cell of origin [12]. Based on this, similarities at the molecular level among different cancer types that are related in terms of histology or anatomy could be used to develop a universal approach for the analysis of cancer; such an analysis is highly important in developing strategies for future therapeutic development [13].

DNA methylation in combination with liquid biopsy is very powerful in identifying circulating epigenetic biomarkers of clinical importance. A recent study has shown that DNA methylation analysis in ctDNA could provide information on the tissue of origin, thus contributing to the improvement of survival of cancer patients for a variety of tumors [14]. Compared to other classes of molecular biomarkers, such as mRNA and proteins, DNA methylation has many advantages since it is chemically stable and can be detected in clinical samples that are kept for a long time even under nonideal storage conditions [15]. Moreover, the incidence of aberrant methylation of specific CpG islands is high in tumor samples and can thus be easily detected by using genome‐wide screening technologies, in contrast to cancer‐specific mutations, which are not only rare but are also spread at many different positions in tumor DNA [15].

Despite all these advantages, up to now clinical tests are commercially available only for a very low number of DNA methylation‐based biomarkers (only 14 at present) [16, 17], and even a lower number of specific DNA methylation‐based tests are approved by the Food and Drug Administration (FDA) or European Medicines Agency as *in vitro* diagnostic (IVD) tests [7].

Changes in DNA methylation patterns in plasma are known to arise early during cancer pathogenesis [18]. Blood‐based DNA methylation tests are thus now being explored to develop tests for early cancer diagnosis [19]. ctDNA methylation analysis was used to stratify patients with primary central nervous system tumors [20] and successfully profile melanoma progression to brain metastasis [21]. According to a recent study, DNA methylation profiling could be used for a histomolecular stratification of patients with brain metastases [22].

Large‐scale targeted methylation sequencing of plasma cfDNA has a high potential for early cancer diagnosis. Recently, a novel assay targeting 9223 CpG sites that are consistently hypermethylated according to TCGA was developed and validated in plasma cfDNA from patients with advanced colorectal cancer (CRC), non‐small‐cell lung carcinoma (NSCLC), breast cancer, and melanoma [23]. According to the results presented, this method could not only detect these types of cancer with high accuracy, but, moreover, methylation scores in plasma cfDNA were correlated with treatment outcomes [23].

Another recent study has shown that cfDNA methylation analysis in plasma could provide some patterns that can detect and discriminate common primary intracranial tumors [24]. In renal cell carcinoma, a novel assay based on a combination of methylated cfDNA immunoprecipitation and high‐throughput sequencing was shown to detect early‐stage tumors and classify patients across all stages of the disease [25]. Based on a sensitive, immunoprecipitation‐based methodology for DNA methylation analysis of cfDNA, tumor‐specific changes were revealed and used further for early cancer detection and classification [26]. It was also shown that methylation profiling has the potential to track evolutionary changes in ctDNA [27].

In this review, we summarize the latest findings on DNA methylation markers in ctDNA for early detection, prognosis, minimal residual disease (MRD), risk of relapse, treatment selection, and resistance, for breast, prostate, lung, and CRC.

## 2 DNA methylation markers in ctDNA

**Fig. 1:**
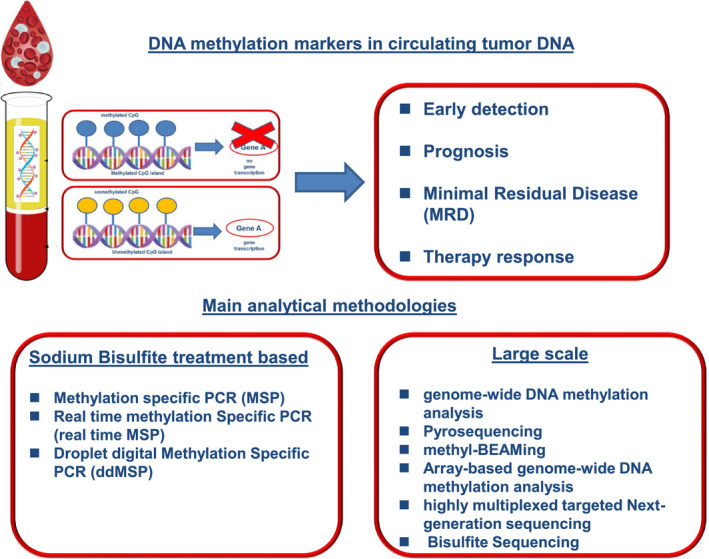
ctDNA methylation markers can provide information on early detection, prognosis, MRD, and therapy response. The main analytical methodologies are based either (a) on PCR following SB conversion or (b) on a large‐scale omic approach.

The main analytical methodologies used for the analysis of ctDNA methylation markers are based either (a) on PCR following sodium bisulfite (SB) treatment that converts all nonmethylated cytocines to uracils and subsequently through PCR to thymines, which include methylation‐specific PCR (MSP), real‐time MSP, and droplet digital MSP (ddMSP) or (b) on a large‐scale omic approaches that include genome‐wide DNA methylation analysis, pyrosequencing, methyl‐BEAMing, array‐based genome‐wide DNA methylation analysis, highly multiplexed targeted next‐generation sequencing, and bisulfite sequencing. ctDNA methylation markers can provide information on early detection, prognosis, MRD, and therapy response in various types of cancer (Fig. 1).

### 2.1 Breast cancer

#### 2.1.1 Early detection

Detection of methylated ctDNA in the peripheral blood of cancer patients is highly promising for the discovery of highly sensitive and specific molecular biomarkers for screening and early detection. Recently, by using genome‐wide DNA methylation analysis, novel cfDNA biomarkers that could differentiate breast cancer patients from healthy individuals were identified for sporadic breast cancer [28]. According to a recent review based on 14 studies, cfDNA methylation analysis is highly promising for both early detection and disease relapse [29]. Methylation of *SPAG6*, *NKX2‐6*, *ITIH5,* and *PER1* genes was detected early in serum of breast cancer patients [30].

Toward the same direction, another study has evaluated the prognostic significance of promoter methylation of seven genes (*APC*, *BRCA1*, *CCND2*, *FOXA1*, *PSAT1*, *RASSF1A,* and *SCGB3A1*) in primary tissues and cfDNA of breast cancer patients [31]. According to the results reported, methylation of *APC*, *FOXA1*, *RASSF1A*, or *SCGB3A1* could discriminate noncancerous from cancerous tissues with an accuracy of 95%. The same study has shown that high *PSAT1* methylation levels were associated with longer disease‐free survival (DFS), and that high *FOXA1* methylation levels were associated with shorter DFS, while a combination of *APC*, *FOXA1*, and *RASSF1A* methylation provided a sensitivity, specificity, and accuracy of over 70% [31].

According to a very recent epigenome‐wide study, the DNA methylation profile in blood starts to change when breast cancer becomes invasive, and these changes can be detected years before the tumor is clinically detected, since differences in DNA methylation patterns are a consequence and not the cause of breast cancer [32]. Early detection of primary breast cancer through the analysis of epigenetic biomarkers in cfDNA by droplet digital PCR (ddPCR) was shown to give accurate results, comparable to mammography screening [33].

#### 2.1.2 Prognosis

*SOX17* promoter methylation was detected in plasma ctDNA in patients with operable breast cancer, after surgical removal of the primary tumor [34]. DNA methylation of five cancer‐related genes (*KLK10*, *SOX17*, *WNT5A*, *MSH2*, and *GATA3*) was evaluated as a prognostic marker in a study that included 150 and 16 breast cancer patients under adjuvant and neoadjuvant therapy, respectively, 34 patients with metastatic disease and 35 healthy volunteers by quantitative MSP (QMSP) [35]. According to the results presented in this study, *GATA3* methylation was detected in all patient groups but not in the healthy control population. In the metastatic setting, *WNT5A* methylation was correlated with poor prognosis and a shorter overall survival (OS), *SOX17* methylation to shorter PFS and OS, and *KLK10* methylation to relapse. When at least 3 or 4 of these genes were methylated, a shorter OS and no response to therapy were documented in this group [35]. Genome‐wide methylation and QMSP have shown that *CCND2* promoter hypermethylation was detected in 40.9% of breast tumors and 44.4% of plasma circulating cfDNA of patients, and could serve as a potential diagnostic and prognostic marker in breast cancer [36].

#### 2.1.3 Minimal residual disease

In operable breast cancer, there is a high risk of recurrence and disease progression after surgery and therapeutic treatment. Early detection of MRD during a disease‐free follow‐up period would greatly improve patient outcomes. However, MRD monitoring in breast cancer through DNA methylation markers in ctDNA analysis is still not well established [37]. In breast cancer, ctDNA assays for mutation detection are not as yet ideal for MRD detection since different mutations can be present in the same gene in individual tumors, while DNA methylation markers of specific genes are universally present in cells of a common type and easier to be identified. In a very recent study, the detection and quantification of breast‐specific DNA methylation patterns in cfDNA were shown to have a sensitivity of 80% and a specificity of 97% for the detection of localized disease; an increase in cfDNA levels was associated with aggressive disease and a decrease was detected after neoadjuvant chemotherapy, while the detection of cfDNA after the completion of chemotherapy was an indication of MRD [38].

#### 2.1.4 Therapy response and resistance

In 25–30% of hormone‐positive metastatic breast cancer patients treated with endocrine therapy, adaptive mechanisms emerge through alterations in the estrogen receptor ligand‐binding domain [39, 40]. Recently, *ESR1* methylation in CTCs and corresponding plasma ctDNA was shown to be a potential biomarker for response to endocrine therapy; moreover, a high concordance of *ESR1* methylation in CTCs and paired plasma ctDNA was shown in the metastatic setting [39]. Another similar study verified these results, by showing that *ESR1* epigenetic status, as evaluated by methylation‐specific ddPCR (MS‐ddPCR), is indicative of resistance to endocrine therapy [40]. TBCRC 005, a multisite prospective study, revealed the prognostic significance of a panel of 10 cfDNA methylation markers for survival outcomes in metastatic breast cancer. However, the clinical utility of this assay for risk stratification and disease monitoring should be further confirmed [41]. In triple‐negative breast cancer, results of the GeparSixto trial have shown that *MGMT* promoter methylation was not related to different chemotherapy response rates [42].

In Table 1, we summarize the main studies on ctDNA methylation markers in breast cancer.

**Table 1:** DNA methylation markers in breast cancer.

DNA methylation markers evaluated	Type of sample/number of patients/controls	DNA methylation markers—of clinical significance	Methodology	Ref
Early detection				
38 differentially methylated CpG positionsSelected marker: CYFIP1	Leukocytes/sporadic breast cancer: 22 + 80/healthy women: 10 + 80	Methylation at *CYFIP1* was identified as a novel epigenetic biomarker candidate for sporadic breast cancer	Genome‐wide DNA methylation analysis Illumina methylation arrays	[28]
*SPAG6, NKX2‐6, ITIH5, PER1*	cfDNA serum test cohort (*n* = 103), serum validation cohort (*n* = 368), plasma cohort (*n* = 125)	Novel biomarker candidates: *SPAG6, NKX2‐6,* and *PER1* DCIS: *SPAG6* and *ITIH5* showed 63% sensitivity for early invasive tumor (pT1, pN0): *SPAG6* and *ITIH5* showed 51% sensitivity and 80% specificity DCIS detection—serum validation cohort: *NKX2‐6* and *ITIH5*, 50% sensitivity DCIS detection—plasma cohort: *SPAG6, PER1,* and *ITIH5* 64% sensitivity	TCGA/human methylation 450 BeadChip data/pyrosequencing	[30]
*APC, BRCA1, CCND2, FOXA1, PSAT1, RASSF1A, and SCGB3A1*	cfDNA cohort #1, *n* = 137 cfDNA cohort #2, *n* = 44	*APC, FOXA1, RASSF1A, and SCGB3A1* discriminated normal from cancerous tissue with high accuracy (95.55%) High *PSAT1* methylation levels associated with longer DFS Higher *FOXA1* methylation levels associated with shorter DFS *APC, FOXA1,* and *RASSF1A* in cfDNA disclosed a sensitivity, specificity, and accuracy over 70%.	Multiplex QMSP	[31]
9601 CpG markers were identified associated with invasive breast cancer	Prospectively collected blood DNA samples from the Sister Study 1552 cases, 224 subcohort	DNA methylation profile in blood starts to change when breast cancer gets invasive	Epigenome‐wide study using Infinium HumanMethylation 450 Bead Chips	[32]
*JAK3, RASGRF1, CPXM1, SHF, DNM3, CAV2, HOXA10, B3GNT5, ST3GAL6, DACH1, P2RX3*, and *chr8:23572595*	Breast tissues: 56 microdissected cfDNA: 34 cell lines, and 29 blood samples from healthy volunteers (HVs, control group) cfDNA samples from 80 HVs and 87 cancer patients	The best detection model adopted four methylation markers (*RASGRF1, CPXM1, HOXA10, and DACH1*) and two parameters (cfDNA concentration and the mean of 12 methylation markers) The area under the receiver operating characteristic curve for cancer normal discrimination was 0.916 and 0.876 in the training and validation dataset, respectively	Array‐based genome‐wide DNA methylation analysis	[33]
	An independent dataset of 53 HVs and 58 BC patients.	The sensitivity and the specificity of the model were 0.862 (stages 0‐I 0.846, IIA 0.862, IIB‐III 0.818, metastatic BC 0.935) and 0.827, respectively Early detection of primary breast cancer through the analysis of epigenetic biomarkers was shown to give accurate results, comparable to mammography screening	cfDNA: ddMSP	
Prognosis				
*SOX17*	79 primary breast tumors, 114 paired samples of DNA isolated from CTCs 114 samples of cfDNA, 60 healthy individuals	There was a significant correlation between SOX17 methylation in cfDNA and CTCs in patients with early breast cancer (*P* = 0.008), but not in patients with verified metastasis (*P* = 0.283) *SOX17* promoter is highly methylated in primary breast tumors, in CTCs isolated from patients with breast cancer, and in corresponding cfDNA samples	MSP	[34]
*KLK10, SOX17, WNT5A, MSH2, GATA3*	Plasma cfDNA: 150 breast cancer patients under adjuvant therapy	The methylation of WNT5A was statistically significantly correlated with greater tumor size and poor prognosis characteristics and in advanced‐stage disease with shorter OS	Quantitative MSP	[35]
	16 breast cancer patients under neoadjuvant therapy	In the metastatic group, also SOX17 methylation was significantly correlated with the incidence of death, shorter PFS, and OS		
	34 patients with metastatic disease	KLK10 methylation was significantly correlated with unfavorable clinicopathological characteristics and relapse, whereas in the adjuvant group to shorter DFI		
	35 healthy volunteers	Methylation of at least 3 or 4 genes was significantly correlated with shorter OS and no pharmacotherapy response, respectively		
CCND2	93 tumors and paired adjacent normal tissues of breast cancer patients circulating cfDNA : 18 breast cancer patients	40.9% of breast tumors 44.4% of plasma circulating cfDNA CCND2 promoter hypermethylation is an independent poor prognostic factor	Genome‐wide methylation and QMSP	[36]
Ten cfDNA methylation markers	Serum samples from 141 women at baseline, at week 4, and at first restaging	Prognostic significance for survival outcomes in metastatic breast cancer	Quantitative multiplex assay (cMethDNA)	[41]
Therapy response				
*ESR1*	CTCs, ctDNA: 65 primary breast tumors FFPE, EpCAM^+^ CTC fractions (122 patients and 30 healthy donors; HD), plasma ctDNA (108 patients and 30HD) CTCs, CellSearch, and paired plasma ctDNA for 58 patients with breast cancer	*ESR1* methylation was detected in: 25/65 (38.5%) FFPEs EpCAM^+^ CTC fractions: 26/112 (23.3%) patients and 1/30 (3.3%) HD Plasma ctDNA: 8/108 (7.4%) patients and 1/30 (3.3%) HD ESR1 methylation was highly concordant in 58 paired DNA samples, isolated from CTCs (CellSearch) and corresponding plasma *ESR1* methylation was observed in 10/36 (27.8%) CTC‐positive samples and was associated with lack of response to treatment (*P* = 0.023, Fisher's exact test)	Real‐time MSP	[39]
*ESR1*	ctDNA: 49 women with hormone receptor‐positive HER2‐negative MBC were prospectively enrolled before treatment start and after 3 months	An epigenetic characterization strategy based on ctDNA is capable of being integrated in the current clinical workflow to give useful insights on treatment sensitivity	MS‐ddPCR	[40]

### 2.2 Prostate cancer

#### 2.2.1 Early detection

Numerous diagnostic tests based on prostate‐specific antigen (PSA) have been developed so far to improve early detection of prostate cancer; however, minimally invasive tests with better specificity and sensitivity are still needed to further improve diagnosis and risk stratification. Tests based on DNA methylation markers in cfDNA are highly promising for early detection since they are minimally invasive, highly specific, and can be detected at very early stages of the disease. These epigenetic alterations can be detected in both plasma and urine of prostate cancer patients [43]. In localized and *de novo* metastatic prostate cancer, three genes, *DOCK2*, *HAPLN3*, and *FBXO30*, were found to be specifically hypermethylated in prostate cancer tissues using MS‐ddPCR [44]. In plasma cfDNA, methylation of these three genes was detected in 61.5% of metastatic prostate cancer patients and was associated with significantly shorter time to progression to metastatic castration‐resistant prostate cancer (mCRPC), indicating a potential usefulness for the identification of hormone‐naïve metastatic prostate cancer patients who could benefit from intensified treatment [44]. It was also recently reported that DNA methylation in regions of chromosome 8q24 may be associated with the risk of developing prostate cancer [45]. Another study has shown that the detection of promoter methylation of *MCAM*, *ERα*, and *ERβ* genes in serum ctDNA could be utilized as a combined biomarker for the early detection of prostate cancer, with a sensitivity and specificity almost equal to and better than serum PSA, respectively [46].

The quantification of DNA methylation levels of another set of eight genes (*APC*, *FOXA1*, *GSTP1*, *HOXD3*, *RARβ2*, *RASSF1A*, *SEPT9,* and *SOX17*) in plasma, based on a multiplex QMSP, was evaluated for the early detection of lung, prostate, and CRC [47]. Out of these eight genes, only two, *SEPT9* and *SOX17*, were methylated in all three cancers; *FOXA1*, *RARβ2,* and *RASSF1A* methylation was detected in lung and prostate cancer with 64% sensitivity and 70% specificity; and methylation of *GSTP1* and *SOX17* could discriminate lung cancer from prostate cancer with 93% specificity [47]. A robust validation of these studies in large prospective cohorts would be of high importance since positive findings could reduce the numbers of unnecessary prostate biopsies.

#### 2.2.2 Prognosis

In localized intermediate‐grade prostate cancer, current clinically established prognostic markers, such as PSA, lack sensitivity and specificity in distinguishing aggressive from indolent disease; toward this direction, the identification of novel prognostic methylation biomarkers for prostate cancer is highly important [48]. In a recent prospective study, the prognostic significance of promoter methylation of *GSTP1* and *APC* in ctDNA was evaluated in castration‐resistant prostate cancer. According to the results reported, pretreatment detection of DNA methylation of these markers is prognostic for worse OS [49]. In another study, DNA methylation of eight genes was evaluated by pyrosequencing in prostate cancer and results were used in relation to the Gleason score for patient stratification. According to the results presented, DNA methylation based on five genes in relation to the Gleason score could predict metastatic lethal progression and is highly promising for risk stratification of patients in the advanced stage [50].

#### 2.2.3 Minimal residual disease

Promoter methylation of two genes, namely *ST6GALNAC3* and *ZNF660*, after being evaluated in 705 prostate cancer tissues and 110 nonmalignant tissue samples, was reported to be cancer‐specific with an area under curve in ROC curves of 0.917–0.995 and 0.846–0.903 [51]. In the same study, hypermethylation of ZNF660 was significantly associated with biochemical recurrence, indicating a potential utility for the stratification of low/intermediate‐grade cases into indolent or more aggressive subtypes [51]. In the same study, using ddPCR, promoter methylation of these two genes (*ST6GALNAC3* and *ZNF660*) and additionally *CCDC181* and *HAPLN3* was evaluated in ctDNA of 27 patients with prostate cancer and 10 patients with benign prostate hyperplasia using MS‐ddPCR [51]. ctDNA analysis based on methylation of three of these genes (*ST6GALNAC3*, *CCDC181*, and *HAPLN3*) could detect prostate cancer with 100% diagnostic specificity and 67% diagnostic sensitivity [51].

#### 2.2.4 Therapy resistance and response

DNA methylation is highly important in prostate cancer initiation and progression. Epigenetic alterations that regulate prostate cancer progression could provide a source of biomarkers indicating resistance or response to specific therapies [52]. Abiraterone acetate (AA) is administered to patients with mCRPC, and the identification of predictive biomarkers to this drug is highly important. A recent study that was performed in plasma cfDNA in a group of 108 samples from 33 prostate cancer patients treated with AA provided a list of DNA methylation‐based predictive biomarkers for response to AA treatment [53]. When serially collected cfDNA samples from mCRPC patients under androgen deprivation therapy were analyzed by genome‐wide methylation analysis, maintenance of changes in DNA methylation profiles indicated a longer time to clinical progression, while the detection of DNA methylation markers for neuroendocrine CRPC indicated a faster time to clinical progression [54]. A recently developed cfDNA methylation assay specific for prostate cancer, called mDETECT, was evaluated in a relatively low number of plasma samples sequentially collected from prostate cancer patients who were beginning androgen deprivation therapy; this test was based on a highly multiplexed targeted next‐generation sequencing of PCR products, comprising 46 PCR probes to 40 regions [53]. Results of this test were comparable to PSA values at different time points [55].

In Table 2, we summarize the main studies on ctDNA methylation markers in prostate cancer.

**Table 2:** DNA methylation markers in prostate cancer.

DNA methylation markers tested	Type of sample/number of patients/controls	Selected DNA methylation markers of clinical significance	Methodology	Ref
Early detection				
DNA methylome	Four metastatic treatment‐naïve prostate cancer (PCa) patients urine and plasma	Urine and plasma are viable surrogates for tumor tissue biopsies, capturing up to 39.40% and 64.14% of tumor‐specific methylation alterations, respectively	Infinium® Methylation EPIC BeadChip (Illumina)	[43]
63 CpG sites located nearby the cancer susceptibility SNPs at 8q24 or in promoter, exon 2, exon 3, or 30 regions for MYC	694 prostate cancer cases including 172 aggressive cases (stage III/IV or Gleason score >8) 516 nonaggressive cases (stage I/II and Gleason score >8) 703 controls	8q24 DNA methylation levels may be associated with prostate cancer risk 8 CpG sites whose DNA methylation levels were associated with the risk of overall prostate cancer The most significant CpG site overall was located at Chr8:128428897 in POU5F1B When the cases were stratified by disease aggressiveness, two moderately correlated CpG sites in MYC (Chr8:128753187 and Chr8:128753154) were identified that were specifically associated with the risk of aggressive but not nonaggressive prostate cancer	Targeted pyrosequencing assays	[45]
*SSBP2, MCAM, ERα, ERβ, APC, CCND2, MGMT, GSTP1, p16, and RARβ2*	84 serum samples from PC, 30 controls 7 cases diagnosed as high‐grade prostatic intraepithelial neoplasia	*MCAM, ERα, and Erβ* combined biomarker for the early detection of prostate cancer, a combination marker panel of *MCAM, ERα,* and *ERβ* increased the sensitivity to 75% and the specificity became 70% for the minimally invasive early detection test of PC with a sensitivity and specificity almost equal and better than serum PSA	QMSP	[46]
Prognosis				
*DOCK2, HAPLN3,* and *FBXO30*	cfDNA plasma samples 36 healthy controls 61 benign prostatic hyperplasia (BPH) 102 localized PCa 65 *de novo* mPCa patients	ctDNA methylation of DOCK2, HAPLN3, and/or FBXO30 was detected in 61.5% (40/65) of *de novo* mPCa patients ctDNA methylation of DOCK2, HAPLN3, and/or FBXO30 was markedly increased in high‐volume compared to low‐volume mPCa (89.3% (25/28) vs 32.1% (10/31), *P* < 0.001) Detection of methylated ctDNA was associated with significantly shorter time to progression to metastatic castration‐resistant PCa, independent of tumor volume Methylated ctDNA (DOCK2/HAPLN3/FBXO30) may be potentially useful for identification of hormone‐naïve mPCa patients who could benefit from intensified treatment.	MS‐ddPCR/cfDNA	[44]
*APC, FOXA1, GSTP1, HOXD3, RARβ2, RASSF1A, SEPT9,* and *SOX17*	Circulating cfDNA 121 PCa 136 asymptomatic donors' plasma samples	*FOXA1, RARβ2, and RASSF1A* detected PCa with 64% sensitivity and 70% specificity	Multiplex QMSP/cfDNA plasma	[47]
*GSTP1 and APC*	Plasma cfDNA prospective study, 50 CRPC patients Control group 10 healthy age‐matched men 10 men aged under 35 10 healthy women	Prognostic significance, OS	MSP	[49]
CpG methylation of eight biomarkers previously identified using the HumanMethylation 450 array	Training dataset: 366 men with no evidence of recurrence and 58 who developed metastasis or died of PCa Testing dataset: 29 cases with metastatic lethal PCa Comparison group: 29 cases who remained recurrence‐free for at least 5 years postsurgery	Five CpGs in relation to the Gleason score could predict metastatic lethal progression and is highly promising for risk stratification of patients in the advanced stage	Pyrosequencing	[50]
MRD				
*ST6GALNAC3* and *ZNF660* in primary tissues *ST6GALNAC3, ZNF660, CCDC181,* and *HAPLN3* promoter methylation in liquid biopsies	705 prostate cancer tissues, 110 nonmalignant tissue samples Liquid biopsies (serum): 27 patients with prostate cancer 10 patients with BPH (control)	In tissues, hypermethylation of *ST6GALNAC3* and *ZNF660* was highly cancer‐specific with AUC of ROC curve analysis of 0.917–0.995 and 0.846–0.903, respectively	Primary tissues: MS qPCR or methylation array Liquid Biopsies: ddMSP analysis	[51]
Therapy response				
485 577 cytosines interrogated by the microarray in each sample	Plasma cfDNA in a group of 108 samples from 33 prostate cancer patients treated with AA	AA differentially modified positions: 26 874 cytosines were differentially modified when comparing AA‐sensitive with the AA‐resistant patients DNA methylation‐based predictive biomarkers for response to AA treatment	Infinium HumanMethylation 450K BeadChip	[53]
cfDNA methylome analysis	45 plasma cfDNA serially collected cfDNA samples from 16 mCRPC patients under androgen deprivation therapy (12 enzalutamide‐treated 4 abiraterone‐treated)	*RUNX3, RGS12,* and *FBP1* *GSTP1, TBX15, AOX1* *NPBWR1, ZSCAN12, PCDHGA11, PHOX2A, TBX10, TEX28, TKTL1,* and *TSPAN32* *B3GNTL1, FAM19A5, INPP5A, MAD1L1, MCF2L, MYT1L,* and *PRDM16* Monitoring the cfDNA methylome during therapy in mCRPC may serve as predictive marker of response to androgen targeting agents	Genome‐wide methylation analysis (cfMeDIP‐seq)	[54]
40 regions were identified that were each methylated between 47% and 94% of TCGA patient samples	Seven patients with biochemical recurrence that were initiating androgen deprivation therapy (ADT)	Overall 86% of patients were positive for 20 or more of these regions, and only 6.6% of patients had 5 or less probes positive mDETECT levels seemed to anticipate rising PSA levels, suggesting it may be able to provide an earlier indication of tumor progression, as well as tracking tumor burden in a PSA‐negative tumor	mDETECT: highly multiplexed targeted next‐generation sequencing of targeted PCR products comprising of 46 PCR probes to 40 regions	[55]

### 2.3 Lung cancer

During the last 20 years, numerous studies have shown the importance of epigenetic alterations in lung cancer. DNA methylation of specific genes has been shown to play an important role in lung cancer pathogenesis, and can provide novel biomarkers for early detection, prognosis, and prediction of response to specific treatments [56]. In parallel, liquid biopsy analysis has a significant contribution to the management of lung cancer patients [57] and the detection of EGFR mutations in plasma cfDNA is now used on a routine basis for the stratification of NSCLC patients [58, 59]. Beyond gene mutations, alterations in DNA methylation of specific genes can provide novel epigenetic biomarkers in NSCLC that could be used for early detection, prognosis, and prediction of response to specific therapies [9, 60, 61]. Detection of aberrant promoter hypermethylation of tumor suppressor genes in serum DNA from NSCLC patients was first reported in 1999 by Esteller *et al*. [62]. Since then, DNA methylation was shown in many studies to be an ideal source of candidate biomarkers since it is stable, easy to detect, and can be detected at high percentages in tumor samples [9, 63]. In lung cancer, a variety of genes were found to be methylated in various types of samples, including tissues, plasma, sputum, and even bronchoscopic washings/brushings [9]. Liquid biopsy analysis of DNA methylation markers is highly promising for diagnosis, prognosis, risk assessment, and disease monitoring [64].

#### 2.3.1 Early detection

Plasma‐based detection of cancer‐specific DNA methylation markers may provide a simple cost‐effective method for the early detection of lung cancer and can be used for noninvasive diagnostics and monitoring. A very recent review on the main approaches to develop biomarkers for the early detection of lung cancer, with considerations of detection of rare tumor events, focused on DNA methylation‐based detection in plasma and sputum [65]. In a recent study, analysis of DNA methylation markers in plasma of NSCLC patients using DNA methylation‐specific qPCR could distinguish lung cancer patients from healthy controls with high sensitivity and specificity [66].

#### 2.3.2 Prognosis

When DNA methylation of *APC*, *HOXA9*, *RARβ2*, and *RASSF1A* was evaluated by QMSP in lung cancer, it was found that *HOXA9* and *RASSF1A* had higher methylation levels in small‐cell lung cancer than in NSCLC, with *HOXA9* methylation levels displaying a sensitivity of 63.8%, and *RASSF1A* displaying a specificity of 96.2% for small‐cell lung cancer detection in ctDNA [67]. Additionally, *HOXA9* methylation levels were higher in squamous cell carcinoma than in adenocarcinoma [67]. Very recently, the detection of *KMT2C* (*MLL3*) promoter methylation was detected in plasma cfDNA of NSCLC patients at both early and advanced stages, but not in plasma of healthy individuals [68]. Promoter methylation of this gene in plasma cfDNA needs to be further evaluated in a large and well‐defined patient cohort [68]. Promoter methylation of two other genes, namely *BRMS1* and *SOX17*, in plasma ctDNA from NSCLC patients has also been shown to be of prognostic significance [69, 70].

#### 2.3.3 Therapy response and resistance

Up to now, only a few studies have evaluated the potential of DNA methylation markers in ctDNA of lung cancer patients for therapy resistance. Recently, a combined detection of somatic mutations and DNA methylation markers in plasma cfDNA was used to evaluate response to osimertinib in NSCLC patients positive for the T790M EGFR mutation [71]. According to the results presented, DNA methylation levels were significantly higher in the plasma samples of patients with somatic mutations than in patients without mutations and healthy controls; a decrease in DNA methylation levels was associated with better treatment efficacy, while an increase indicated disease progression [71].

In Table 3, we summarize the main studies on ctDNA methylation markers in lung cancer.

**Table 3:** DNA methylation markers in lung cancer.

DNA methylation markers tested	Type of sample/number of patients/controls	Selected DNA methylation markers of clinical significance	Methodology	Ref
Early detection				
Set of 10 marker loci	Liquid biopsy test plasma cfDNA NSCLC patients: 18 healthy: 47	Distinguish lung cancer patients from healthy controls with high sensitivity and specificity	Real‐time MSP	[66]
*APC, HOXA9, RARβ2, RASSF1A*	152 tissue samples 129 plasma samples; 28 benign lesions of lung	*HOXA9* and *RASSF1A* displayed higher methylation levels in SCLC than in NSCLC *HOXA9* methylation levels sensitivity: 63.8% for SCLC detection in ccfDNA *RASSF1A* methylation levels specificity: 96.2% for SCLC detection in ccfDNA	Quantitative MSP	[67]
*KMT2C* (MLL3)	Operable NSCLC: 48 fresh frozen NSCLC tissues, 48 adjacent non‐neoplastic tissues, 48 matched plasma samples Metastatic NSCLC: 91 NSCLC plasma samples; 60 plasma samples from HD	In metastatic NSCLC, *KMT2C* promoter methylation in plasma cfDNA was related to worse PFS worse OS	Real‐time MSP	[68]
Prognosis				
*HOXA9, KRTAP8‐1, CCND1, TULP2*	TCGA: 338 tissue samples from lung adenocarcinoma patients including 149 nonmalignant ones Tumor samples and matched adjacent lung samples from 25 patients	Methylation of HOXA9, KRTAP8‐1, CCND1, and TULP2 has great potential for the early recognition of lung adenocarcinoma	Pyrosequencing	[60]
*MGMT, p16, DAP kinase, GSTP1*	Normal lung, primary NSCLC, and corresponding serum were obtained from each of the 22 patients	First report on the detection of aberrant promoter hypermethylation of tumor suppressor genes Abnormal promoter hypermethylation of tumor suppressor genes is readily detectable in the serum DNA of cancer patients using MSP analysis	MSP	[62]
*SOX17*	Operable NSCLC: 57 primary tumors and paired adjacent noncancerous tissues and in ctDNA isolated from 48 corresponding plasma samples Advanced NSCLC: Plasma from 74 patients with and 49 healthy individuals	Detection of SOX17 promoter methylation in plasma provides prognostic information	Real‐time MSP	[69]
Breast cancer metastasis suppressor 1 (BRMS1)	57 NSCLC tumors and adjacent noncancerous tissues, cfDNA, 48 corresponding plasma samples, cfDNA isolated from plasma of 74 patients with advanced NSCLC and 24 healthy individuals.	Methylation of *BRMS1* promoter in cfDNA isolated from plasma of NSCLC patients provides important prognostic information	Real‐time MSP	[70]
Therapy response				
Combined detection of somatic mutations and DNA methylation markers	85 longitudinal plasma samples obtained from 8 stage IV osimertinib‐treated EGFR T790 M‐positive lung adenocarcinoma patients	The methylation levels were significantly higher in the plasma samples of patients with detectable somatic mutations than patients without somatic mutations and healthy controls. A decrease in DNA methylation levels was associated with the efficacy of treatment, while an increase was indicating disease progression	Bisulfite sequencing	[71]

### 2.4 Colorectal cancer

#### 2.4.1 Early detection

In CRC, a blood‐based test based on real‐time MSP detection of methylated Septin9 in DNA obtained from peripheral blood samples has been FDA‐approved for early detection; however, a positive result should still be verified by colonoscopy or sigmoidoscopy [17]. Methylated Septin9 also has a high potential to be used as a routine biomarker for CRC recurrence monitoring, especially in combination with contrast‐enhanced computed tomography [72]. Another approach to identify potential serum methylation biomarkers for the detection of advanced CRC is to use pooled samples; unsupervised clustering has shown that cfDNA methylation patterns can distinguish advanced neoplasia from healthy controls [73]. Methylated ctDNA markers are highly promising for the development of a blood‐based CRC screening liquid biopsy test [74, 75, 76].

#### 2.4.2 Prognosis

*RASSF1A* promoter methylation was reported as a prognostic biomarker in patients with stage II and III CRC receiving oxaliplatin‐based chemotherapy, when investigated by MSP in 108 CRC patients before and after chemotherapy and 78 healthy controls [77]. DNA methylation of *BCAT1* and *IKZF1* was recently reported in ctDNA of CRC patients, and it was shown to be related to CRC stage; after surgery, these DNA methylation markers were not detected, indicating a possible role of these markers on the adequacy of surgical resection [78].

#### 2.4.3 Therapy response and resistance

In a prospective study, a combined detection of *NPY* methylation along with tumor‐specific mutations in ctDNA could give similar results to radiographic evaluation, showing that this combined liquid biopsy approach can be used for the follow‐up of mCRC patients during treatment [79]. According to a recent study analysis, a five‐gene methylation panel (*EYA4*, *GRIA4*, *ITGA4*, *MAP3K14* *‐AS1,* and *MSC*) in cfDNA using ddPCR can be used in cases where patient‐specific mutations cannot be detected for monitoring tumor burden dynamics in liquid biopsy under different therapeutic regimens [80]. The detection of MGMT methylation in plasma ctDNA could be used as a predictive biomarker of response to alkylating agents [81].

In Table 4, we summarize the main studies on ctDNA methylation markers in CRC.

**Table 4:** DNA methylation markers in CRC.

DNA methylation markers tested	Type of sample/number of patients/controls	Selected DNA methylation markers of clinical significance	Methodology	Ref
Early detection				
Septin9	93 patients with CRC and 94 individuals with no evidence of disease 135 patients with CRC, 91 healthy controls, 169 patients with adenomatous polyps 81 patients with hyperplastic polyps	FDA‐approved for early detection of CRC a positive result should still be verified by colonoscopy or sigmoidoscopy discriminated between patients with CRC and healthy controls with high clinical sensitivity and specificity in pivotal case–control studies	Real‐time MSP Epi proColon® 2.0 CE	[17]
Septin9	650 plasma samples	Routine biomarker for CRC recurrence monitoring, especially in combination with contrast‐enhanced computed tomography mSEPT9 analysis might be popularized as a routine biomarker for CRC screening. The combined detection of mSEPT9 and CECT can play an important role for recurrence monitoring	Real‐time MSP Epi proColon® 2.0 CE	[72]
866 836 CpG positions across the genome	Serum samples from 20 individuals with no colorectal findings, 20 patients with advanced adenomas, 20 patients with CRC (stages I and II)	cfDNA methylation patterns can distinguish advanced neoplasia from healthy controls The differential methylation analysis revealed 1384 CpG sites with at least 10% difference in the methylation level between no colorectal findings controls and advanced neoplasia, the majority of which were hypomethylated	Methylation levels of 866 836 CpG positions across the genome using the MethylationEPIC array Unsupervised clustering	[73]
Prognosis				
*RASSF1A*	108 CRC patients before and after chemotherapy and 78 healthy controls	Promoter methylation of RASSF1A can influence sensitivity to oxaliplatin‐based chemotherapy, which can be used to predict outcomes for patients with stage II and stage III CRC	Real‐time MSP	[77]
MRD				
*BCAT1* and *IKZF1*	91 cancer tissues 187 cfDNA samples	Significant methylation of either BCAT1 or IKZF1 was seen in 86/91 (94.5%) cancer tissues ctDNA methylated in BCAT1 or IKZF1 was detected in 116 (62.0%) cases at diagnosis and was significantly more likely to be detected with later stage and distal tumor location BCAT1 and IKZF1 related to CRC stage while after surgery these DNA methylation markers were not detected, indicating a possible role of these markers on the adequacy of surgical resection	Real‐time multiplex PCR assay	[78]
Therapy response				
*NPY*	24 metastatic CRC patients	Prospective study, a combined detection of NPY methylation along with tumor‐specific mutations in ctDNA could give similar results to radiographic evaluation, showing that this combined liquid biopsy approach can be used for the follow‐up of mCRC patients during treatment	Droplet‐based digital PCR (ddPCR)	[79]
*EYA4, GRIA4, ITGA4, MAP3K14* *‐AS1, MSC*	85 tissue DNA 182 cfDNA from mCRC patients	87% of mCRC patients (87%) showed positivity in at least one marker *EYA4*: 67%, *GRIA4*: 71.3%, *ITGA4*: 69.2%, *MAP3K14* *‐AS1*: 69.8%, *MSC*: 62.1% Methylation can be used as a universal test to circumvent the absence of patient‐specific mutations for monitoring tumor burden dynamics *via* liquid biopsy	Genome‐wide methylation microarrays ddPCR	[80]
*MGMT*	60 metastatic CRC tissue samples	Predictive biomarker of response to alkylating agents	Methyl‐BEAMing Pyrosequencing	(81)

## 3 ctDNA methylation assays: standardization and pre‐analytical considerations

Pre‐analytical conditions could significantly affect the results of DNA methylation analyses in liquid biopsies. For this reason, standardization of pre‐analytical conditions and implementation of quality control steps is extremely important for reliable liquid biopsy analysis, and a prerequisite for routine applications in the clinic. In a recent systematic study, the stability of DNA methylation in plasma and SB‐converted DNA under different storage conditions was evaluated [63]. In the same study, the reliability of whole‐genome amplification procedures for SB‐converted DNA samples was checked by real‐time MSP for *ACTB*, *SOX17,* and *BRMS1*. According to this study, plasma and SB‐converted DNA samples are stable and can be used safely for MSP when kept at −80 °C [63].

Different blood collection tubes and cfDNA isolation methods can influence the cfDNA amount and the detection of promoter methylation [82, 83]. Recently, there has been an international effort to standardize liquid biopsy procedures and protocols, such as the International Liquid Biopsy Standardization Alliance (https://fnih.org/what‐we‐do/biomarkers‐consortium/programs/ilsa)—who recently provided a white paper focused on the independent liquid biopsy‐ and standardization‐based programs [84]—the European Liquid Biopsy Society (https://www.uke.de/english/departments‐institutes/institutes/tumor‐biology/european‐liquid‐biopsy‐society‐elbs/index.html) and the International Society of Liquid Biopsy (https://www.isliquidbiopsy.org/).

## 4 Conclusions and future perspectives

ctDNA methylation analysis has the potential to improve early cancer detection, which could lead to a substantial reduction in cancer‐related mortality. Biomarkers based on DNA methylation in ctDNA have a huge potential to be used for screening, early diagnosis, and in predicting and monitoring the response to specific therapies [85]. However, despite considerable interest in the field of discovering and developing novel biomarkers, a lot of improvements are still necessary. Toward the discovery of novel reliable biomarkers that can be measured with high specificity and sensitivity at an acceptable cost in the routine clinical setting, epigenetic alterations in liquid biopsy samples are highly promising. However, further research is required to determine which of these methylated ctDNA markers are the most accurate when applied to large cohorts of patients.
